# Supplementary material for: A Randomized Controlled Trial of Soy Isoflavone Intake on Mammographic Density among Malaysian Women
Source: Nutrients. 2023 Jan 6;15(2):299. doi: 10.3390/nu15020299 (PMC9862880; doi:10.3390/nu15020299)
Supplement: Supplementary file 1 [file nutrients-15-00299-s001.zip › nutrients-2079398-supplementary.pdf]

Supplementary Table S1. Comparison of characteristics between participants who completed and those who were lost in follow up

Characteristics	All (n = 118)	Distribution by study completion		p Value
		Completed (n = 91)	Lost in follow up (n = 27)	
Demographic				
Age in years, median (IQR)	57 (6.8)	57 (6.5)	57 (6.5)	0.785
Ethnicity, n (%)				
Chinese	92 (78.0)	72 (79.1)	20 (74.1)	0.683
Indian	16 (13.6)	11 (12.1)	5 (18.5)	
Malay	10 (8.5)	8 (8.8)	2 (7.4)	
Education, n (%)				
Up to secondary	34 (28.8)	28 (30.8)	6 (22.2)	0.623
Tertiary	81 (68.6)	62 (68.1)	19 (70.4)	
Monthly household income, n (%)				
<RM5,000	42 (35.6)	33 (36.3)	9 (33.3)	0.999
RM5,000 – 10,000	38 (32.2)	29 (31.9)	9 (33.3)	
> RM10,000	33 (28.0)	26 (28.6)	7 (25.9)	
Reproductive				
No. of children, n (%)				
None	24 (20.3)	17 (18.7)	7 (25.9)	0.612
1-2	52 (44.1)	41 (45.1)	11 (40.7)	
> 3	39 (33.1)	32 (35.2)	7 (25.9)	
Age at last menstrual period, median (IQR)	51.0 (6.0)	51.0 (5.2)	50.5 (5.8)	0.138
Years since last menstrual period, n (%)				
Less than 5 years	51 (43.2)	38 (41.8)	13 (48.1)	0.654
5 years or more	63 (53.4)	50 (54.9)	13 (48.1)	
Use of oral contraceptives, n (%)	34 (28.8)	29 (31.9)	5 (18.5)	0.323
Family history & screening				
1° Family history, n (%)				
Any cancer	53 (44.9)	42 (46.2)	11 (40.7)	0.665
Breast cancer	15 (12.7)	11 (12.1)	4 (14.8)	0.746

Last mammogram, *n* (%)				
1-2 years	34 (28.8)	28 (30.8)	6 (22.2)	0.781
More than 2 years	79 (66.9)	59 (64.8)	20 (74.1)	
Never	5 (4.2)	4 (4.4)	1 (3.7)	
Lifestyle factors at enrolment				
BMI, kg/m^2, *n* (%)				
< 25 (low)	73 (61.9)	53 (58.2)	20 (74.1)	0.177
≥ 25 (high)	45 (38.1)	38 (41.8)	7 (25.9)	
Physical activity, MET-hours/week, *n* (%)				
≤ 10 (low)	59 (50.0)	46 (50.5)	13 (48.1)	0.999
> 10 (moderate/high)	55 (46.6)	42 (46.2)	13 (48.1)	
Soy isoflavone intake, mg/day, median (IQR)	17.8 (21.2)	17.8 (22.9)	16.9 (21.6)	0.096
Mammographic density at enrolment, median (IQR)				
Dense area, cm^2	15.1 (20.3)	14.6 (18.5)	19.2 (22.5)	0.066
Percent density, %	13.3 (22.7)	12.7 (21.8)	21.0 (19.9)	0.033

Supplementary Table S2. Change in area-based percent mammographic density over the study period (n = 90)

	n	Change in percent mammographic density, median (IQR)			
		Absolute change (%)	p Value	Relative change (%)	p Value
By study arm					
All completed participants					
ISF Supplement	27	-1.4 (6.4)	0.347	-13.9 (45.9)	0.999
ISF Diet	30	-0.5 (3.6)		-11.8 (45.6)	
Control	33	-1.1 (5.4)		-16.4 (53.8)	
< 5 years since menopause					
ISF Supplement	10	-4.4 (7.1)	0.061	-22.8 (30.1)	0.227
ISF Diet	14	-0.7 (3.7)		-15.2 (44.4)	
Control	13	-0.8 (6.5)		-14.4 (37.7)	
≥ 5 years since menopause					
ISF Supplement	16	-0.4 (3.5)	0.307	-9.1 (41.1)	0.660
ISF Diet	15	-0.3 (3.1)		-11.5 (44.8)	
Control	19	-1.5 (4.2)		-27.7 (51.3)	
By total isoflavone intake					
All completed participants					
<18mg/day	23	-0.8 (4.2)	0.441	-20.2 (60.4)	0.615
18-61mg/day	22	-2.0 (6.2)		-14.8 (57.5)	
61-101mg/day	21	-1.3 (2.9)		-11.6 (32.5)	
>101mg/day	24	-0.2 (6.7)		-10.4 (54.5)	
< 5 years since menopause					
<18mg/day	11	-0.8 (5.8)	0.411	-13.9 (36.1)	0.285
18-61mg/day	9	-3.3 (9.2)		-10.2 (46.9)	
61-101mg/day	8	-1.2 (4.4)		-21.2 (33.2)	
>101mg/day	9	-3.5 (8.9)		-18.3 (25.4)	
≥ 5 years since menopause					
<18mg/day	11	-1.1 (2.1)	0.100	-36.9 (45.1)	0.282
18-61mg/day	12	-1.9 (4.6)		-20.8 (52.7)	
61-101mg/day	13	-1.3 (2.1)		-11.5 (36.5)	
>101mg/day	14	1.1 (3.5)		7.3 (46.5)	

Supplementary Table S3. Change in dense volume over the study period (*n* = 90)

	n	Change in dense volume, median (IQR)			
		Absolute change (cm^3)	*p* Value	Relative change (%)	*p* Value
By study arm					
All completed participants					
ISF Supplement	24	-0.9 (6.8)	0.550	-1.3 (19.4)	0.544
ISF Diet	29	-2.0 (8.1)		-4.2 (16.3)	
Control	32	-1.8 (10.4)		-5.5 (22.9)	
< 5 years since menopause					
ISF Supplement	10	-0.9 (6.3)	0.328	-2.4 (12.6)	0.328
ISF Diet	14	-3.7 (7.4)		-9.4 (11.5)	
Control	13	0.3 (9.5)		0.5 (21.9)	
≥ 5 years since menopause					
ISF Supplement	16	0.3 (7.3)	0.503	0.4 (20.5)	0.380
ISF Diet	15	0.2 (6.8)		0.3 (16.9)	
Control	19	-1.9 (9.2)		-6.4 (17.8)	
By total isoflavone intake					
All completed participants					
<18mg/day	22	0.2 (7.0)	0.671	0.4 (15.7)	0.535
18-61mg/day	21	-1.9 (13.2)		-5.1 (26.7)	
61-101mg/day	20	0.6 (8.8)		0.6 (22.2)	
>101mg/day	22	-2.2 (4.9)		-4.9 (12.6)	
< 5 years since menopause					
<18mg/day	11	-2.8 (6.8)	0.390	-7.4 (15.8)	0.373
18-61mg/day	9	0.8 (12.0)		2.5 (23.0)	
61-101mg/day	8	-3.1 (6.0)		-8.3 (10.9)	
>101mg/day	9	-2.9 (23.9)		-6.6 (24.7)	
≥ 5 years since menopause					
<18mg/day	11	1.3 (5.3)	0.192	2.5 (11.2)	0.177
18-61mg/day	12	-4.0 (8.4)		-9.9 (13.4)	
61-101mg/day	13	2.2 (10.4)		7.7 (25.8)	
>101mg/day	14	-2.3 (3.9)		-5.5 (8.8)	

Supplementary Table S4. Distribution of total soy isoflavone intake at Month 12 by study arm

Total soy isoflavone intake (mg/day)	*n*	Distribution by study arm, *n* (%)		
		ISF Supplements (*n* = 27)	ISF Diet (*n* = 30)	Control (*n* = 33)
<18mg/day	23	4 (14.8)	3 (10.0)	16 (48.5)
18-61mg/day	22	4 (14.8)	4 (13.3)	14 (42.4)
61-101mg/day	21	4 (14.8)	14 (46.7)	3 (9.1)
>101mg/day	24	15 (55.6)	9 (30.0)	0 (0)

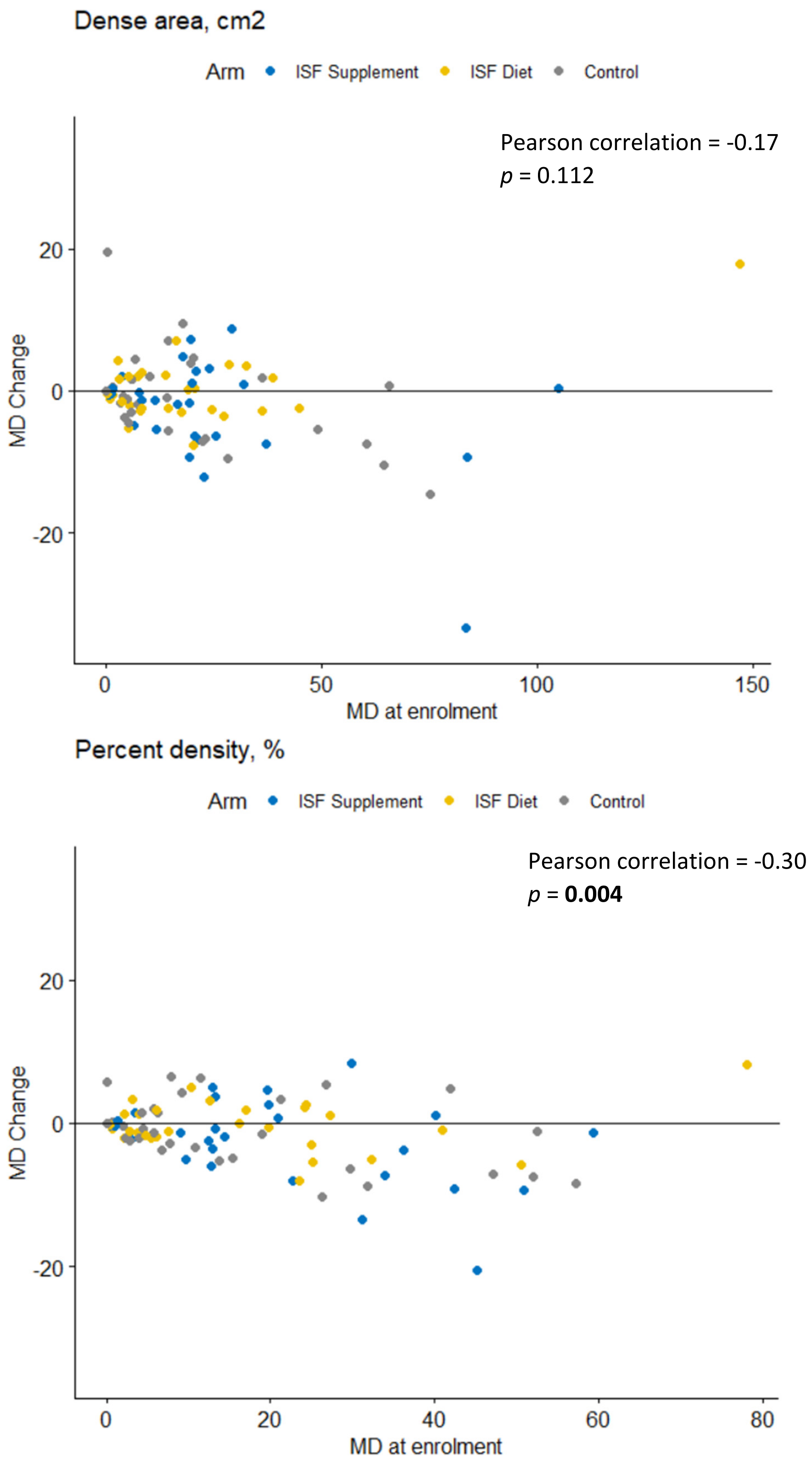

Supplementary Figure S1 Distribution of MD change by MD at enrolment for dense area and percent density
